# Extracellular vesicle-mediated EBAG9 transfer from cancer cells to tumor microenvironment promotes immune escape and tumor progression

**DOI:** 10.1038/s41389-017-0022-6

**Published:** 2018-01-24

**Authors:** Toshiaki Miyazaki, Kazuhiro Ikeda, Wataru Sato, Kuniko Horie-Inoue, Satoshi Inoue

**Affiliations:** 1 Division of Gene Regulation and Signal Transduction, Research Center for Genomic Medicine, Saitama Medical University, Saitama, 350-1241 Japan; 2 Department of Functional Biogerontology, Tokyo Metropolitan Institute of Gerontology, Tokyo, 173-0015 Japan

## Abstract

The antitumor immune response is a critical defense system that eliminates malignant cells. The failure of the system results in immune escape and proceeds to tumor growth. We have previously showed that estrogen receptor-binding fragment-associated antigen 9 (EBAG9) is a relevant cancer biomarker and facilities immune escape of cancers from the immune surveillance. EBAG9 in cancer cells suppresses T-cell infiltration into tumor in vivo, whereas that in host immune cells functions as a limiter for T-cell cytotoxicity. Considering that EBAG9 plays immune suppressive roles in both tumor and microenvironment, we here questioned whether EBAG9 is a transferable protein from cancer to surrounding T cells and affects antitumor immune response. In this study, we showed that spontaneous development of prostate cancer was repressed in a model of *Ebag9* knockout mice crossed with transgenic adenocarcinoma of the mouse prostate (TRAMP) mice. We identified TM9SF1 as a collaborative EBAG9 interactor, which regulates epithelial-mesenchymal transition (EMT) in cancer cells. Notably, extracellular vesicles (EVs) from EBAG9-overexpressing prostate cancer cells have a potential to facilitate immune escape of tumors by inhibiting T-cell cytotoxicity and modulating immune-related gene expression in T cells. Furthermore, we showed that a neutralizing antibody for EBAG9 could rescue the EV-mediated immune suppression by recovering T-cell cytotoxicity. In addition to its autocrine functions in cancer cells, EBAG9 could behave as a new class of immune checkpoint that suppresses tumor immunity in a secretory manner. We propose that EBAG9-targeting cancer treatment could be alternative therapeutic options for advanced diseases, particularly for those with EBAG9 overexpression.

## Introduction

Cancer progression is regulated by interactions between cancer cells and their tissue microenvironment containing immune cells. Malignant cells are eliminated by immune surveillance at first; however, cancer cells will often obtain ability to evade from the immune system, a process known as immune escape^1^. T lymphocytes are primary mediators of the adaptive immune response and play an important role in the tumor surveillance^2,3^. In particular, cytotoxic CD8^+^ T lymphocytes (CTLs) are activated to kill cancer cells through the recognition of specific antigen on the cancer cells by using T-cell receptor (TCR) system. Understanding the mechanisms of tumor immunity are clinically relevant to develop alternative immune therapies^4^.

Estrogen receptor-binding fragment-associated antigen 9 (*EBAG9*) has been originally identified as an estrogen-responsive gene in breast cancer cells^5^. EBAG9 stimulates the migration of cultured cancer cells and clinically associates with pathophysiology of various cancers including breast, ovarian, prostate, hepatocellular, renal, and bladder cancers^6–12^. We previously showed that EBAG9 immunoreactivity was inversely associated with the infiltration of CD3^+^ T cells in breast cancer specimens^6^. In Renca syngeneic transplantation renal cancer models, we demonstrated that EBAG9 overexpression stimulated the tumor formation of syngeneic transplantation model^10^. These findings implied that EBAG9 functions as a tumor-promoting factor through influencing host tissue microenvironment, including immune cells; however, the precise mechanism of cancer-derived EBAG9 remains elusive.

Notably, we recently showed that tumor formation and metastasis of transplanted cancer cells were repressed in *Ebag9* knockout (*Ebag9*KO) mouse host, and CD8^+^ T cells infiltration was significantly enhanced in the transplanted tumors^13^. The tumor-infiltrated CD8^+^ T cells from *Ebag9*KO host exhibited increased degranulation and cytotoxic activity. Moreover, adoptive transfer of transplanted tumor-infiltrated *Ebag9*KO CD8^+^ T cells suppressed inoculated tumor growth in wild-type host. In the model, the cytotoxic activity of *Ebag9*KO CD8^+^ T cells was enhanced as the T cell-derived granzymes were upregulated without increased proliferation of T cells^14^. EBAG9 was also shown to interact with γ2-adaptin, a clathrin adaptor-related protein, which might be involved in endosome-to-lysosome maturation^15^. These findings suggested that host EBAG9 functions as a suppressor for tumor growth and metastasis in tumor microenvironment by repressing the cytotoxicity of immune cells.

Recent advance in cancer research revealed that tumor cells often secret larger amounts of extracellular vesicles (EVs), including exosomes than normal cells into their microenvironment^16,17^. Exosomes are defined as a subset of small extracellular vesicles (30–100 nm diameter) and contain various bioactive molecules including nucleic acids and proteins^18,19^. As exosomes primarily function as mediators in intercellular communication and molecular transfer, a role of exosomes in tumor development and metastasis has attracted considerable attention^20^. In particular, it is notable that tumor-derived exosomes sometimes facilitate immunosuppression by inhibiting the activities of immune cells^21^.

Here we questioned whether EBAG9 or cancer-derived EVs containing EBAG9 affect cancer cell biology, as well as immune surveillance system in tumor microenvironment. In the study of antitumor immune response, we used a model of autochthonous spontaneous transgenic adenocarcinoma of the mouse prostate (TRAMP)^22^. When TRAMP mice were crossed with *Ebag9*KO mice, we showed that prostate tumor development was repressed compared with parental TRAMP mice. We identified a novel interacting factor for EBAG9, transmembrane 9 superfamily member 1 (TM9SF1)^23,24^. TM9SF1 and EBAG9 cooperatively regulate prostate cancer cell migration by modulating the expression of genes involved in epithelial-mesenchymal transition (EMT). Notably, we showed that cancer-derived EVs contain EBAG9 protein, which promotes EMT of prostate cancer cells, as well as represses the cytotoxicity of T cells. EBAG9 monoclonal antibody substantially neutralizes the EV-dependent reduction of T cell-mediated cytotoxicity. We infer that tumor-derived EV-mediated EBAG9 protein is a critical factor that potentially facilitates tumor progression by negatively modulating immune cells in microenvironment.

## Results

### Cancer development is reduced in spontaneous prostate cancer model mice with EBAG9 deficiency

**Fig. 1:**
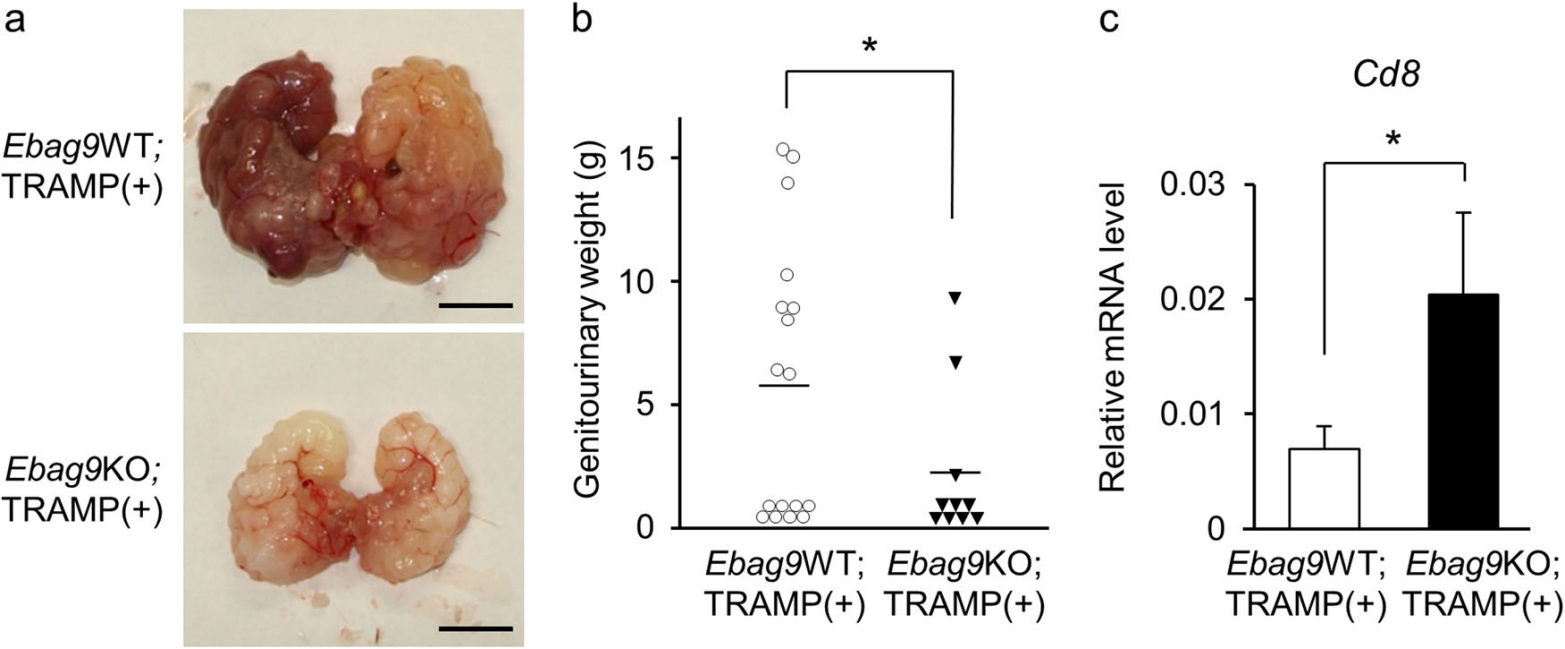
EBAG9 suppresses prostate tumor development in spontaneous transgenic adenocarcinoma of the mouse prostate (TRAMP) model. **a** Representative genitourinary organs. *Ebag9*KO mice were crossed with TRAMP mice to generate *Ebag9*KO;TRAMP(+) and control *Ebag9*WT;TRAMP(+) mice. Genitourinary organs were dissected from these male mice at 1 year old. Scale bar, 1 cm. **b** Prostate tumor development was repressed in *Ebag9*KO;TRAMP(+) mice. Genitourinary organ weight was measured at 1 year old. **c** Upregulation of *Cd8* mRNA in prostate tumors generated in *Ebag9*KO;TRAMP(+) mice. qRT-PCR analysis of *Cd8* mRNA was performed using RNAs from prostate tumors. The data are shown as mean ± SD (*Ebag9*KO;TRAMP(+), *n* = 10; *Ebag9*WT;TRAMP(+), *n* = 17). *P < 0.05 (two-sided Student’s *t*-test)

To assess the physiological function of EBAG9 in host defense against prostate cancer development, we used a model of *Ebag9* knockout (*Ebag9*KO) mice^13^ crossed with TRAMP mice (C57BL/6-Tg(TRAMP)8247Ng/J obtained from Jackson Lab), which are characterized to develop spontaneously transgenic adenocarcinoma of the mouse prostate^22^. TRAMP transgene hemizygous mice harbor aggressive forms of prostate cancer with distant metastasis due to the expression of probasin-driven SV40 T antigen. Whole weights of genitourinary tract organs, including prostate are often used as an indicator for monitoring tumor progression^22^. As a control strain for *Ebag9*KO;TRAMP(+) mice, we also generated *Ebag9*WT;TRAMP(+) mice. *Ebag9*KO;TRAMP(+) male mice showed decreased genitourinary weight compared with *Ebag9*WT;TRAMP(+) mice at 1 year after birth (Fig. 1a, b). To evaluate infiltrating T cells in the whole tumor, qRT-PCR was performed using RNAs prepared from the generated tumors^25–27^. It was notable that the mRNA expression of *Cd8*, a selective marker for cytotoxic T cells, was significantly increased in prostate cancer tissues derived from *Ebag9*KO;TRAMP(+) mice compared with control mice (Fig. 1c). These results suggest that host EBAG9 could facilitate cancer development by inhibiting CD8^+^ T-cell infiltration.

### EBAG9 expression in cancer cells promotes cell migration by affecting EMT-related gene levels

**Fig. 2:**
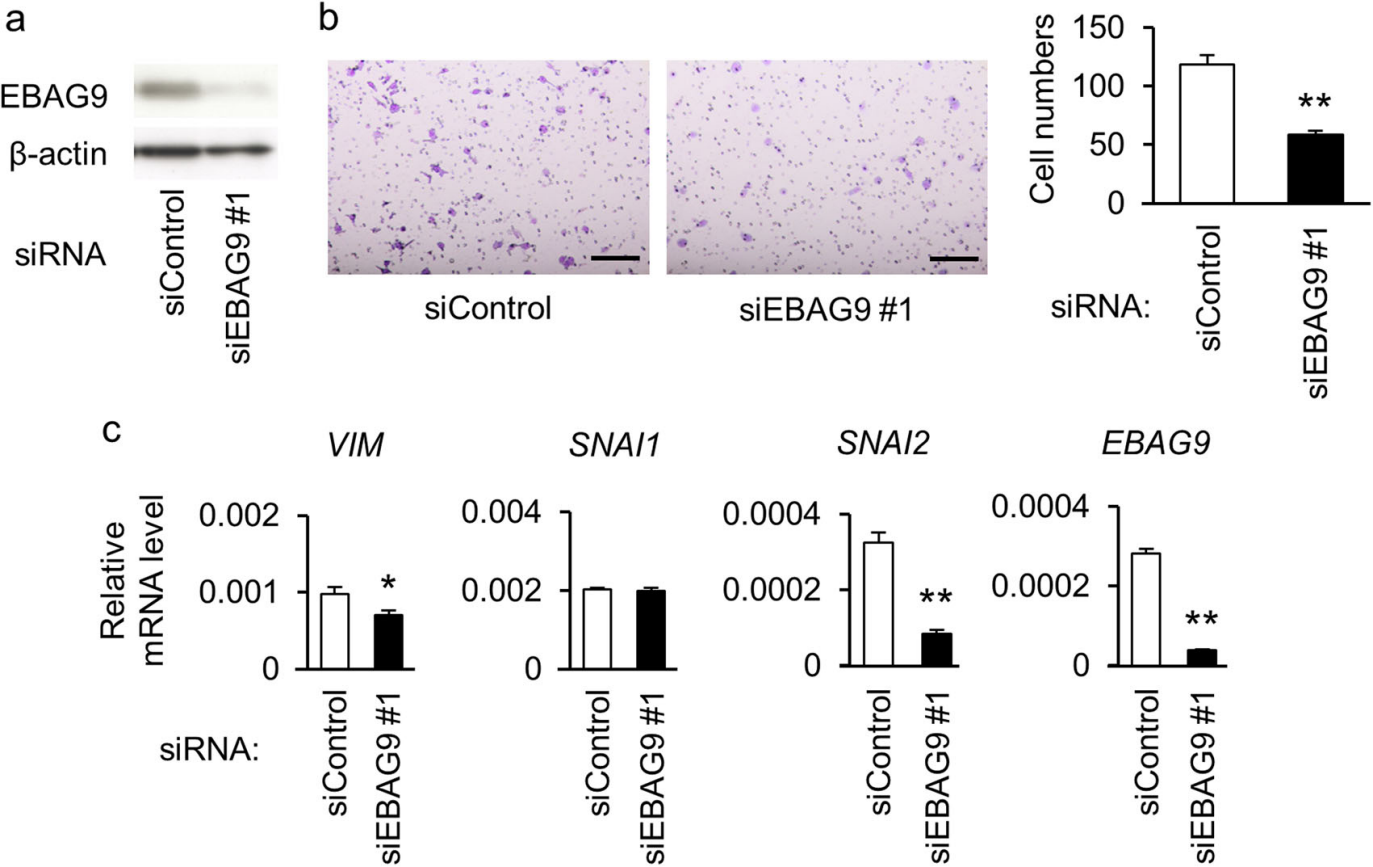
EBAG9 silencing suppresses cancer cell migration and modulates EMT-related gene expression. **a** EBAG9 silencing decreases EBAG9 protein expression in LNCaP cells. The cells were transfected with siRNA targeting EBAG9 (siEBAG9 #1) or non-targeting control siRNA (siControl). **b** siEBAG9 #1 inhibits LNCaP cell migration. Cells transfected with indicated siRNAs were seeded on the upper chamber and migrated cells were stained after 48 h. Representative images of migrated cells are shown. Scale bar, 20 μm. Migrated cell numbers were counted in 5 microscopic fields at least. Data are shown as mean ± SD (*n* = 5). **c** Effect of EBAG9 knockdown on EMT-related gene expression in LNCaP cells. qRT-PCR analyses for *VIM*, *SNAI1*, *SNAI2*, and *EBAG9* mRNA were performed using RNAs prepared from LNCaP cells treated with siEBAG9 #1 or siControl. Data are shown as mean ± SD (*n* = 3). *P < 0.05; **P < 0.01 (two-sided Student’s *t*-test)

**Fig. 3:**
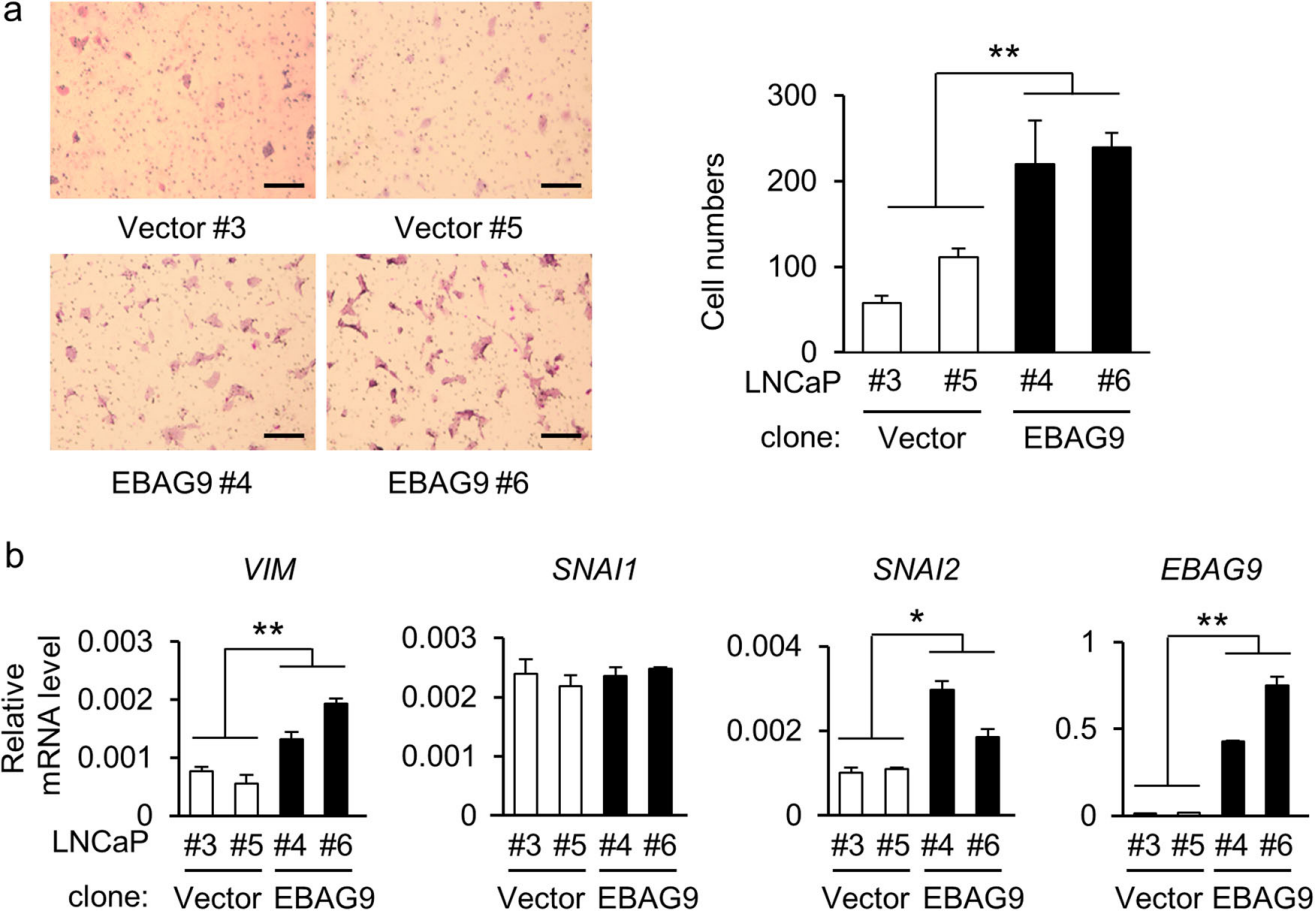
EBAG9 overexpression increases the migration of prostate cancer cells and modulates EMT-related gene expression. **a** EBAG9 increases LNCaP cell migration. Method is described in Fig. 2b. Scale bar, 20 μm. The effect of EBAG9 overexpression on the migration of LNCaP cells is quantitated by counting the migrated cells in at least 5 fields under the microscope. The data are shown as mean ± SD (*n* = 5). **b** Regulation of EMT-related genes in LNCaP-EBAG9 cells. qRT-PCR analyses of *VIM*, *SNAI1*, *SNAI2*, and *EBAG9* mRNA were performed using RNAs prepared from LNCaP-EBAG9 #4 and #6 and LNCaP-Vector #3 and #5 cells. Data are shown as mean ± SD (*n* = 3). *P < 0.05; **P < 0.01 (Statistical analysis was performed using the two-sided Mann–Whitney *U*-test)

To investigate the role of EBAG9 in the pathophysiology of prostate cancer cells, we performed functional studies using siRNA targeting EBAG9 (siEBAG9) in androgen-sensitive LNCaP and androgen-refractory DU145 cells. Western blot analysis showed that siEBAG9 substantially suppressed EBAG9 protein expression in LNCaP cells (Fig. 2a). siEBAG9 significantly inhibited the migration of LNCaP cells (Fig. 2b and Supplementary Figure S1A) and DU145 cells (Supplementary Figure S2A). Decrease of EBAG9 mRNA in DU145 cells treated with siEBAG9 was confirmed by qRT-PCR (Supplementary Figure S2B). We examined the expression of epithelial-mesenchymal transition (EMT)-associated genes as cancer cell migration is often associated with EMT^28^. siEBAG9 decreased the expression levels of a mesenchymal marker, *VIM* mRNA and protein, and an EMT-related transcription factor, *SNAI2* mRNA, in LNCaP cells (Fig. 2c and Supplementary Figures S1B and C). From the viewpoint of EBAG9 overexpression, LNCaP cells stably expressing EBAG9 (LNCaP-EBAG9) exhibited the increases of migration (Fig. 3a) compared with control cells transfected with empty vector (LNCaP-Vector). In addition, *VIM* at mRNA and protein levels and *SNAI2* at mRNA level, were upregulated in LNCaP-EBAG9 cells (Fig. 3b and Supplementary Figure S3). The gain- and loss-of-function studies of EBAG9 indicate that EBAG9 could modulate the gene expression associated with EMT, which may contribute to prostate cancer progression.

### TM9SF1 interacts with EBAG9 and regulates EMT in prostate cancer cells

**Fig. 4:**
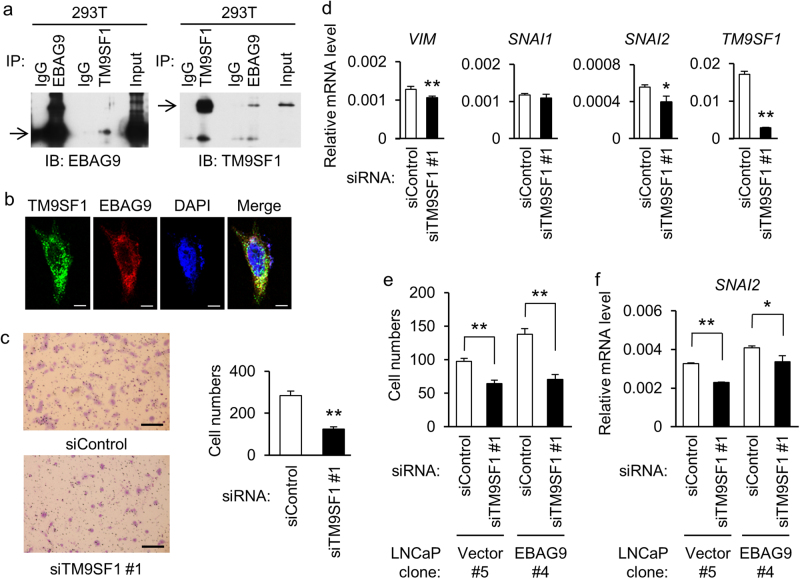
EBAG9 interacts with TM9SF1 to regulate migration and EMT in prostate cancer cells. **a** Interaction between EBAG9 and TM9SF1 in 293T cells. Lysate of 293T cells transfected with Flag-EBAG9 and HA-TM9SF1 plasmids was immunoprecipitated with anti-EBAG9, anti-TM9SF1 or control IgG, then subjected to western blot analysis using EBAG9 (left panel) or TM9SF1 (right panel) antibody. Arrows show signals for Flag-EBAG9 and HA-TM9SF1. **b** Subcellular co-localizatiion of EBAG9 and TM9SF1. HeLa cells were transfected with GFP-TM9SF1 and DsRed-EBAG9, and subjected to fluorescent microscopic examination. Scale bars, 10 μm. **c** TM9SF1 silencing inhibits LNCaP cell migration. Cells transfected with siRNA targeting TM9SF1 (siTM9SF1 #1) or siControl were seeded on the upper chamber and migrated cells were stained after 48 h. Representative images are shown. Scale bar, 20 μm. Migrated cell numbers were counted in 5 microscopic fields at least. Data are shown as mean ± SD (*n* = 5). **d** TM9SF1 modulates EMT-related gene expression in LNCaP cells. qRT-PCR analyses for indicated genes were performed using RNAs prepared from LNCaP cells treated with siTM9SF1 #1 or siControl. **e** TM9SF1 contributes to cell migration interacting with EBAG9. LNCaP-EBAG9 #4 and LNCaP-Vector #5 cells were transfected with siTM9SF1 #1 or siControl, and cell migration was examined as described above. Data are shown as mean ± SD (*n* = 5). **f** TM9SF1 plays a role in EMT-related gene expression along with EBAG9. LNCaP-EBAG9 #4 and LNCaP-Vector #5 cells were transfected with siTM9SF1 #1 or siControl, and *SNAI2* mRNA expression was quantified as described. Data are shown as mean ± SD (*n* = 3). *P < 0.05; **P < 0.01 (two-sided Student’s *t*-test)

To clarify the molecular mechanism of EBAG9, we explored its interacting partners by yeast two-hybrid screening and identified several candidate proteins that are associated with cellular functions of molecular chaperone, fatty acid metabolism, ubiquitin modification, and protein secretion. We focused on transmembrane 9 superfamily member 1 (TM9SF1), whose protein co-localizes with lysosomes in cytosol and plays a role in autophagy^23^. We examined whether EBAG9 physically associates with TM9SF1. 293T cells were cotransfected with HA-TM9SF1 and Flag-EBAG9. Immunoprecipitation was carried out using antibodies to EBAG9 or TM9SF1, and immunoblots of the immunoprecipitates were further detected with anti-EBAG9 and anti-TM9SF1 antibodies. As shown in Fig. 4a, EBAG9 interacted with TM9SF1. Cytosolic co-localization of EBAG9 and TM9SF1 proteins was observed fluoromicroscopically in HeLa cells transfected with GFP-TM9SF1 and DsRed-EBAG9 (Fig. 4b). To investigate the role of TM9SF1 in prostate cancer cells, we performed functional studies using siRNA targeting TM9SF1 (siTM9SF1) in LNCaP cells. siTM9SF1 significantly inhibited the migration of LNCaP cells (Fig. 4c and Supplementary Figure S4A). siTM9SF1 also downregulated *VIM* and *SNAI2* expressions in LNCaP cells (Fig. 4d and Supplementary Figure S4B). We further analyzed whether TM9SF1 contributes to EBAG9-dependent increase in cell migration. siTM9SF1 partially impaired the increases in cell migration (Fig. 4e) and SNAI2 expression (Fig. 4f) in EBAG9-overexpressing LNCaP cells. These results suggest that TM9SF1 could cooperatively function with EBAG9 to regulate cell migration and EMT in prostate cancer cells.

### EVs from EBAG9-overexpressing LNCaP cells stimulate parental cell migration

**Fig. 5:**
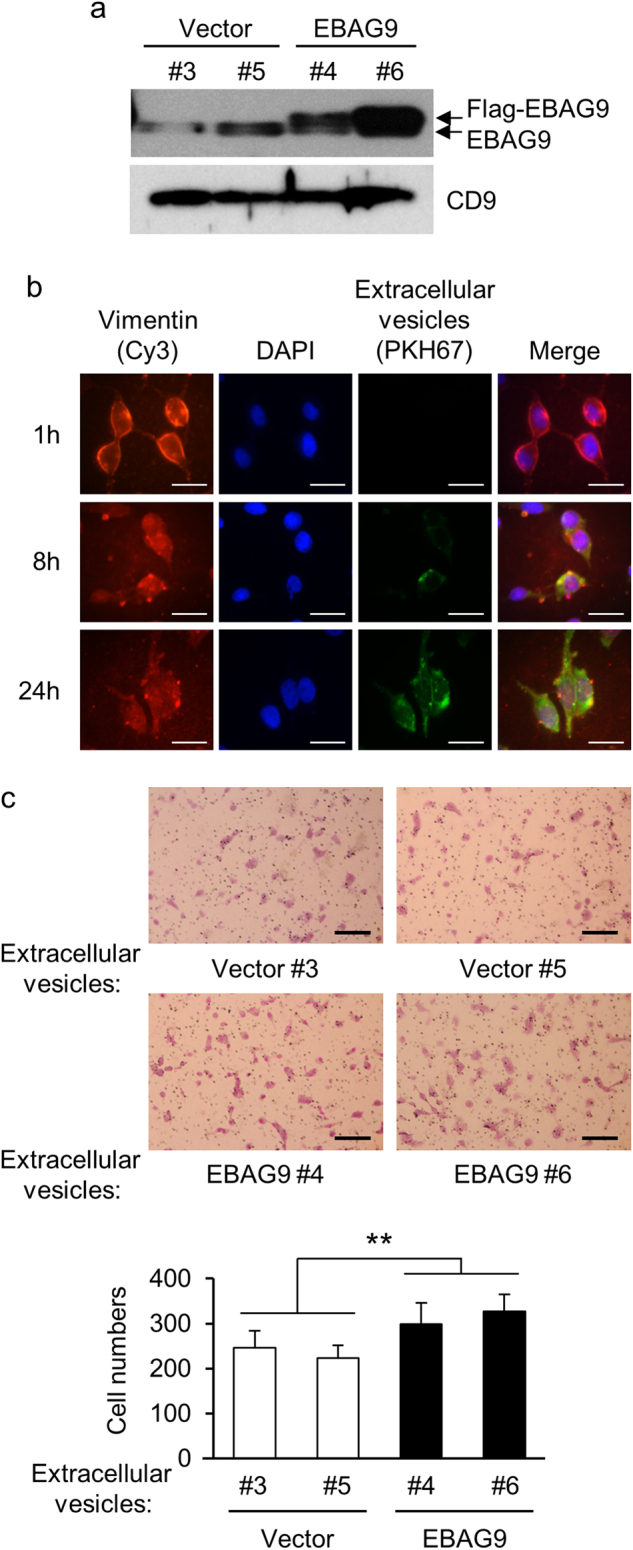
Extracellular vesicle (EV)-transferred EBAG9 stimulates cancer cell migration. **a** EBAG9 protein is secreted in EVs. EVs were prepared from LNCaP-EBAG9 cells (EBAG9 #4 and #6) and control cells (Vector #3 and #5), and subjected to western blot analysis using EBAG9 antibody. **b** EVs are integrated into LNCaP cells. Cells were co-cultured with green fluorescent PKH67-labeled EVs for indicated times and co-stained with Cy3-labeled anti-vimentin antibody and 4′,6-diamidino-2-phenylindole (DAPI). Scale bars, 10 μm. **c** Cancer-derived EVs containing EBAG9 stimulate parental cell migration. LNCaP cells were incubated with LNCaP-EBAG9 cell-derived (EBAG9 #4 and #6) or control cell-derived (Vector #3 and #5) EVs, and cell migration was evaluated as described in Fig. 4. Data are shown as mean ± SD (*n* = 5). **P < 0.01 (Statistical analysis was performed using the two-sided Mann–Whitney *U*-test)

Cancer cells are known to secrete various factors which will affect their behavior and tumor microenvironment in autocrine or paracrine manner^29,30^. We questioned whether EBAG9 protein is secreted in EVs to influence cancer microenvironment. Western blot analysis detected endogenous expression of EBAG9 protein in the EVs prepared from conditioned media of LNCaP-Vector and LNCaP-EBAG9 cells (Fig. 5a, lower signals), indicating that EBAG9 is primarily secreted from prostate cancer cells. Exogenously introduced Flag-EBAG9 was detected only in LNCaP-EBAG9 cells (Fig. 5a, upper signals). To investigate EV internalization, LNCaP-EBAG9 cell-derived EVs were labeled with green fluorescent PKH67 and then added into the culture media of parental LNCaP cells (Fig. 5b). PKH67 signal was accumulated in parental cells, indicating that EVs could be transferred into LNCaP cells in autocrine/paracrine manners. Moreover, LNCaP-EBAG9 cell-derived EVs increased the migration of parental cells (Fig. 5c) and DU145 cells (Supplementary Figure S2C) compared with control cell-derived EVs.

### Cancer-derived EBAG9-containing EVs inhibit cytotoxic activity and modulate immune-related gene expression in T cells

**Fig. 6:**
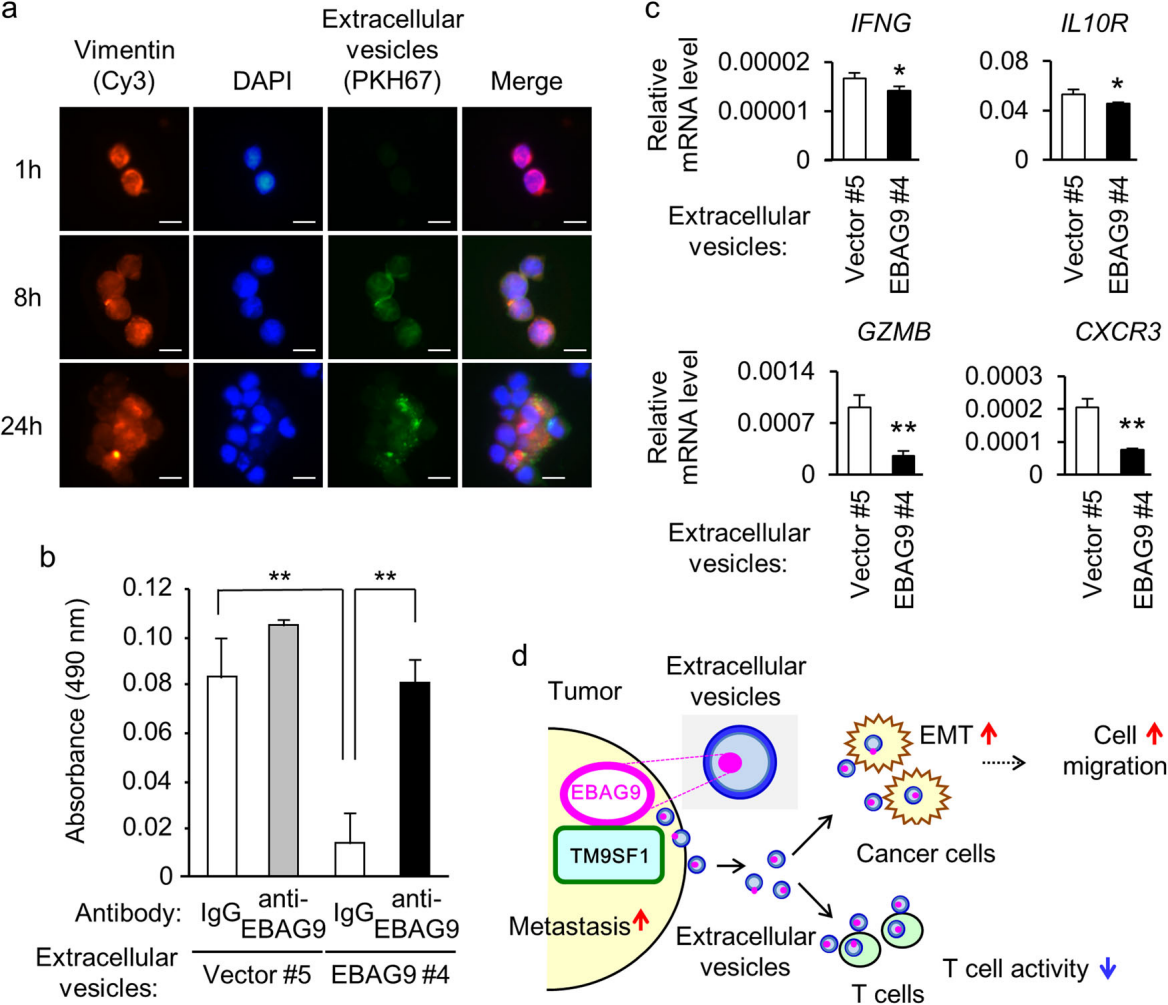
EBAG9-containing extracellular vesicles (EVs) suppress T-cell cytotoxicity. **a** Uptake of EVs into T cells. Fluorescent images of MOLT4 cells were taken at indicated times after co-culture with green fluorescent PKH67-labeled EVs prepared from LNCaP-EBAG9 #4 cells. Cells were co-stained with Cy3-labeled anti-vimentin antibody and 4′,6-diamidino-2-phenylindole (DAPI). Scale bars, 10 μm. **b** EBAG9 monoclonal antibody neutralizes EVs-mediated suppression of T cell cytotoxicity. EVs preincubated with anti-EBAG9 or IgG were washed with PBS and then co-cultured with MOLT4 cells for 2 days. Cytotoxicity was assayed using CytoTox 96 non-radioactive cytotoxicity assay in which lactate dehydrogenase (LDH) released into the media from damaged cells were quantified as absorbance at 490 nm. Data are shown as mean ± SD (*n* = 4). **c** EVs derived from LNCaP-EBAG9 cells downregulate immune-related genes in MOLT4 cells. qRT-PCR analyses for *IFNG*, *IL10R*, *GZMB*, and *CXCR3* expression were performed using RNAs prepared from MOLT4 cells treated with EVs. Data are shown as mean ± SD (*n* = 3). *P < 0.05; **P < 0.01 (two-sided Student’s *t*-test). **d** A proposed model for the EBAG9-dependent regulation of tumor immunity and cancer cell migration in tumor microenvironment. EBAG9 interacts with TM9SF1 in tumor cells and regulates cancer cell migration and EMT. EBAG9-containing EVs also suppress cytotoxicity of T cells involved in microenvironment

We next investigated whether LNCaP-EBAG9 cell-derived EVs affect T cell function. PKH67-labeled EVs from LNCaP-EBAG9 cells were accumulated in T-leukemia MOLT4 cells 8 and 24 h after co-culture treatment (Fig. 6a). Lactate dehydrogenase (LDH) cytotoxicity assay was also performed to evaluate the effects of EBAG9-containing EVs to cytotoxic activity of MOLT4 cells. LNCaP-EBAG9 cell-derived EVs significantly decreased the cytotoxicity of MOLT4 cells against LNCaP cells compared with control cell-derived EVs (Fig. 6b). Interestingly, pretreatment of LNCaP-EBAG9 cell-derived EVs with EBAG9 monoclonal antibody recovered the cytotoxicity of MOLT4 cells to the level equivalent to pretreatment with control IgG, indicating that the antibody could neutralize T cell cytotoxicity. In addition, LNCaP-EBAG9 cell-derived EVs downregulated the expression of immune-related genes containing interferon gamma (*IFNG*), interleukin 10 receptor (*IL10R*), granzyme B (*GZMB*), and chemokine (C-X-C motif) receptor 3 (*CXCR3*) in MOLT4 cells (Fig. 6c). Taken together, we infer that cancer-derived EBAG9-containig EVs affect both on cancer cells and infiltrated T cells by promoting tumor cell migration and suppressing T cell cytotoxicity, respectively (Fig. 6d). We propose that the EBAG9 interactor TM9SF1 may cooperatively function in the EBAG9-mediated tumor-promoting effects.

## Discussion

In this study, *Ebag9*KO mice crossed with TRAMP mice demonstrated that EBAG9 plays a crucial role in prostate cancer development. In prostate cancer cells, EBAG9 regulated cell migration and EMT-related gene expression through the interaction with TM9SF1. In addition, EVs, which contained overexpressed EBAG9 protein, increased the migration of prostate cancer cells. Intriguingly, EBAG9-overexpressing EVs inhibited the cytotoxic activity and immune-related genes expression in MOLT4 cells. These results suggested that cancer-derived EBAG9 would contribute to tumor progression in autocrine and/or paracrine fashions interacting with its novel partner TM9SF1. EBAG9 would transfer to immune T cells through EVs and negatively regulate tumor surveillance in host cells. Another possibility which has not been entirely rules out is that EBAG9 functions through the membrane surface in the recipient cell.

TM9SF1 is a member of the transmembrane 9 superfamily, whose precise function remains unknown. TM9SF1 has been identified by screening genes stimulating autophagosome formation in mammalian cells^23^. TM9SF1 protein is shown to co-localize to lysosomes with LC3, microtubule-associated protein 1A/1B-light chain 3, which is known to form a stable association with the membrane of autophagosomes^23^. TM9SF1 overexpression increases autophagosome formation and actually induces autophagy^23^. It is also shown that TM9SF1 protein is involved in late endosomes and lysosomes^31^. Although TM9SF1 is ubiquitously expressed in various normal tissues, TM9SF1 overexpression is reported in several cancers. In breast cancer, TM9SF1 has been identified by screening cancer specific antigens that are potentially applied to immunotherapy^32^. The screening was specialized for exploring genes encoding functional transmembrane motifs that are differentially expressed between cancer and normal cells. In this study, TM9SF1 was overexpressed in breast cancer tissues compared with normal breast. In urinary bladder cancer, expression microarray analysis revealed that TM9SF1 is one of the genes commonly overexpressed in cancer tissues compared to normal tissues^24^. While the mechanism of TM9SF1 remains to be elusive, we speculate that EBAG9 and TM9SF1 could cooperatively regulate cancer cell migration.

EMT often associates with enhanced cell migration and metastasis, resulting in aggressive cancer phenotypes^33^. Upregulation of vimentin (*VIM*) expression is a typical characteristic of cells undergoing EMT. Several transcription factors, such as SNAIL (*SNAI1*) and SLUG (*SNAI2*), are also known to play crucial roles in EMT^34^. For example, SNAI2 functions as a metastasis regulator in tumors and its expression is associated with poor clinical outcomes for patients with several types of cancer^35^. This study revealed that EBAG9 modulates the expression of EMT-related genes containing *VIM*, *SNAI1*, *and SNAI2* in prostate cancer cells, suggesting that EBAG9 could stimulate cancer progression and invasion through EMT pathway.

It has been reported that EBAG9 is localized in the Golgi apparatus and increases the generation of the tumor-associated *O*-linked glycan Tn (*N*-acetyl-D-galactosamine, GalNAc) and the closely related TF (Thomsen-Friedenreich, Galβ1–3GalNAc) antigens^36^. EBAG9 protein was also shown to localize in the coat protein complex I (COPI)-coated transport vesicles, and EBAG9 negatively regulates the COPI-dependent endoplasmic reticulum-to-Golgi transport pathway and exocytosis^37^. Taken together, EBAG9 would contribute to pathogenesis of cancer cells by inhibiting intracellular membrane trafficking, which results in the alteration of glycosylation modification of cellular proteins, including tumor-associated glyco-antigens^38^. We could speculate that the EBAG9-mediated glycosylation may modify unknown EMT-related proteins in cancer cells. In line with this speculation, atypical glycans undergoing EMT have been identified in cancer cells^39^.

This study showed that cancer-derived EVs contain EBAG9 protein, and proposed that EV-mediated EBAG9 transfer would contribute to cancer cell migration and immune escape. Several studies have reported that tumor-derived EVs can induce T-cell apoptosis^40,41^. For instance, EVs expressing Fas ligand increase T-cell apoptosis in human melanoma and colorectal cancer cells^42,43^. EVs isolated from various cancer cell lines also downregulate natural-killer group 2, member D (NKG2D) expression in NK cells and CD8^+^ cytotoxic MOLT4 cells^44^, where NKG2D is known to function as an activating receptor on the cell surface. In addition, tumor cell-derived EVs modulate inflammatory tumor microenvironment where NF-kappaB signaling plays a critical role^45,46^. We demonstrated that EBAG9 protein is secreted in cancer-derived EVs, which are transferred to cancer cells and T cells, and function as a suppressor for T cell cytotoxicity. The present study provides new insights into tumor progression and anti-tumor immunity, and will provide a potential therapeutic target for advanced cancers.

## Materials and methods

### Cell culture

LNCaP, DU145, 293T, MOLT4, and HeLa cell lines were obtained from the American Type Culture Collection. LNCaP and MOLT4 cells were cultured in RPMI, supplemented with 10% fetal bovine serum (FBS), penicillin (50 U/ml), and streptomycin (50 μg/ml). DU145, 293T, and HeLa cells were cultured in DMEM, supplemented with 10% FBS, penicillin (50 U/ml), and streptomycin (50 μg/ml). LNCaP cells stably expressing flag-tagged EBAG9 (EBAG9 #4 and #6) and control vector (Vector #3 and #5) were selected by G418. Cells were cultured in a humidified atmosphere containing 5% CO_2_ at 37 °C. Short tandem repeat (STR) analyses were performed for the authentication of the cell lines, which were not contaminated with mycoplasma based on the examination by MycoAlert mycoplasma detection kit (Lonza, Walkersville, MD, USA).

### Animal experiments

C57BL/6 TRAMP mice (C57BL/6-Tg(TRAMP)8247Ng/J) were purchased from the Jackson Laboratory and *Ebag9*KO mice (*Ebag*9^−/−^) were described previously^13^. TRAMP mice were crossed with *Ebag9*KO mice to generate *Ebag9*KO;TRAMP(+) mice. At 1 year old, mice were killed and the genitourinary (GU) complex (consisting of prostate, seminal vesicles, urethra, and empty bladder) was weighed. Mice were kept under specific pathogen-free conditions and fed dry food and water ad libitum. The results are shown as mean values ± SD (*Ebag9*KO;TRAMP(+); *n* = 10, *Ebag9*WT;TRAMP(+); *n* = 17). Statistical analysis was carried out using the modified Thompson tau technique and two-sided Student’s *t*-test. *P < 0.05. All experiments were performed according to the guidelines for the care and use of laboratory animals of Saitama Medical University. Animals were randomly assigned to the groups and the experiments were performed without blinding. The sample size estimate was based on our pilot study.

### Small interfering RNA

Small interfering RNAs (siRNAs) of EBAG9 (siEBAG9 #1 and #2) and TM9SF1 (siTM9SF1 #1 and #2) were synthesized by RNAi Inc. (Tokyo, Japan) using an algorithm that significantly improves the target specificity of siRNA, in particular, by efficiently estimating off-target sequences^47^. The specific sequences targeting EBAG9 and TM9SF1 were as follows: siEBAG9 #1 sense 5′-CUCAUUCCUAAAGAGAUUAAU-3′ and antisense 5′-UAAUCUCUUUAGGAAUGAGAA-3′; siEBAG9 #2 sense 5′-AAGAAGAUGCAGCCUGGCAAG-3′ and antisense 5′-UGCCAGGCUGCAUCUUCUUCU-3′; siTM9SF1 #1 sense 5′-GAAUGGCUGAGUCUUUGUAUG-3′ and antisense 5′-UACAAAGACUCAGCCAUUCGG-3′; siTM9SF1 #2 sense 5′-GCCCUGAGAAGAUACGUCACA-3′ and antisense 5′-UGACGUAUCUUCUCAGGGCAG-3′. A non-targeting control siRNA (siControl) with no homology to known gene targets in mammalian cells were also obtained from RNAi Inc.^47^. Transfection of siRNA was carried out using RNAiMAX transfection reagent (Life Technologies, Carlsbad, CA, USA).

### Migration assay

Cell migration was examined by using cell culture inserts with 8.0-μm pore size PET filters (Becton Dickinson Labware, Bedford, MA, USA). Cells were seeded into the culture dish and transfected with 10 nM siEBAG9 #1, siEBAG9 #2, siTM9SF1 #1, siTM9SF1 #2 or siControl using Lipofectamine RNAiMAX reagent (Life Technologies). After transfection, 5 × 10^4^ LNCaP or 3 × 10^4^ DU145 cells were suspended in the culture medium and added to the upper chamber. For stable cell lines (EBAG9 #4 and #6, and Vector #3 and #5), 5 × 10^4^ cells were added to the upper chamber. Cells were then incubated for 24 or 48 h at 37 °C. The cells on the lower surface of the filter were fixed in methanol and stained with Giemsa’s staining solution. After washing, the cells on the lower surface were counted in at least 5 fields under the microscope. The results were shown as mean ± SD (*n* = 5). Statistical analysis was carried out using two-sided Student’s *t*-test. *P < 0.05; **P < 0.01.

### Yeast two-hybrid screen

*Saccharomyces cerevisiae* strain EGY48 was transformed with pEG202-NLS-human EBAG9, and further transformed with a mouse embryo cDNA library (OriGene, Rockville, MD, USA). Screening was carried out according to the manufacturer’s protocol. Positive colonies were selected through growth on agar plates lacking leucine, and verified by galactose-dependent lacZ expression. Plasmids were recovered from the several positive clones and sequenced using the dideoxy termination method.

### Plasmids

The cDNA coding for TM9SF1 was subcloned into pEGFP-N1 expression vector (Clontech, Palo Alto, CA, USA) using *Xho*I and *Hin*dIII restriction sites. The TM9SF1 cDNA was N-terminally tagged with the HA epitope and subcloned into the pcDNA3 vector (Invitrogen, Carlsbad, CA, USA) using *Bam*HI and *Xho*I restriction sites. The cDNA coding for EBAG9 was subcloned into pDsRed-N1 vector (Clontech) using *Xho*I and *Hin*dIII restriction sites. The EBAG9 cDNA was N-terminally tagged with the Flag epitope and subcloned into the pcDNA3 vector using *Eco*RI and *Xho*I restriction sites. Transfection of plasmids was carried out using Lipofectamine 2000 transfection reagent (Life Technologies).

### Western blot analysis and immunoprecipitation

The expression vectors for EBAG9 and TM9SF1 were introduced into 293T cells by transfection for 48 h. Cells were suspended in lysis buffer (20 mM HEPES, pH 7.9, 300 mM NaCl, 1 mM EDTA, 15% glycerol, 0.5% Nonidet P-40, 1 mM Na_3_VO_4_, 1 mM phenylmethylsulfonyl fluoride), rocked gently for 50 min at 4 °C, and centrifuged at 14,000×*g* for 5 min at 4 °C. The supernatants were used as cell lysates. Cell lysates were resolved by SDS-PAGE and electrophoretically transferred onto polyvinylidene fluoride membranes (Millipore, Billerica, MA, USA). The membranes were probed with mouse anti-EBAG9 monoclonal antibody^12^, rabbit anti-CD9 polyclonal antibody (sc-9148, Santa Cruz Biotechnology, Santa Cruz, CA, USA), rabbit anti-vimentin polyclonal antibody (SAB4503081, Sigma-Aldrich, St Louis, MO, USA), or rabbit anti-TM9SF1 polyclonal antibody (APR44683_T100, AVIVA Systems Biology, San Diego, CA, USA). For immuneprecipitation, cell lysates were incubated with the indicated antibody overnight at 4 °C. Then, protein G-sepharose beads (GE Healthcare Japan, Tokyo, Japan) was added to collect the immunocomplexes for an additional 1 h. The pellets were washed three times with lysis buffer and subjected to western blot analysis.

### qRT-PCR

Total RNAs were extracted from prostate cancer LNCaP cells using ISOGEN reagent (Nippon Gene, Toyama, Japan), and first-stand cDNA was generated with 1 μg of total RNA using SuperScript III reverse transcriptase (Life Technologies) and oligo(dT)_20_ primer. To evaluate the expression levels of *Cd8*, *VIM*, *SNAI1*, *SNAI2*, *EBAG9*, *TM9SF1*, *IFNG*, *IL10R*, *GZMB*, and *CXCR3*, quantitative reverse transcription-polymerase chain reaction (qRT-PCR) was performed by StepOnePlus Real-Time PCR system (Life Technologies) using specific primers (sense and antisense, respectively): mouse *Cd8*, 5′- GCTACCACAGGAGCCGAAAG-3′ and 5′-TGGGCTTGCCTTCCTGTCT-3′; human *VIM*, 5′-TGGATTCACTCCCTCTGGTTG-3′ and 5′-CGTGATGCTGAGAAGTTTCGTT-3′; *SNAI1*, 5′-CTAGGCCCTGGCTGCTACAA-3′ and 5′-TCTGAGTGGGTCTGGAGGTG-3′; *SNAI2*, 5′-CGGACCCACACATTACCTTG-3′ and 5′-TGACCTGTCTGCAAATGCTCT-3′; *EBAG9*, 5′-GGGAGCACAGGTTTCTCTAGT-3′ and 5′-CCTGCCAGGTATCTAAGTCAC-3′; *TM9SF1*, 5′-ATGACCTGGCTCGGTACAAC-3′ and 5′-TGTCACCCTGGTCAAAGTCA-3′; *IFNG*, 5′-AGCTCTGCATCGTTTTGGGTT-3′ and 5′-GTTCCATTATCCGCTACATCTGAA-3′; *IL10R*, 5′-GAAGACCGAGGCCATGAGG-3′ and 5′-GGCTGAATTTGCAGATGAGCA-3′; *GZMB*, 5′-CTGATACGAGACGACTTC-3′ and 5′-GGATTATAGGCTGGATGG-3′; *CXCR3*, 5′-GTCCTTGAGGTGAGTGACCA-3′ and 5′-AGCACGAGTCACTCTCGTTT-3′. Human *GAPDH* was used as an internal control^47^.

### EVs isolation and uptake assay

EVs were prepared from the supernatant of LNCaP cells stably expressing EBAG9 or empty vector cultured in media without FBS using supercentrifugation as described^48^.

The EVs were labeled using the PKH67 green fluorescent labeling Kit (Sigma-Aldrich Japan, Tokyo, Japan) according to the manufacturer’s instruction. To examine uptake of PKH67-labeled EVs in LNCaP or MOLT4 cells, cells were incubated with PKH67-labeled EVs for the indicated times and fixed with 4% paraformaldehyde. Cells were costained with Cy3-labeled anti-vimentin antibody (Santa Cruz Biotechnology) and 4′,6-diamidino-2-phenylindole (DAPI).

### Cytotoxicity assay

The EVs prepared from LNCaP cells stably transfected with EBAG9 or empty vector were preincubated with EBAG9 monoclonal antibody^12^ or mouse normal IgG for 2 h at 4 °C. MOLT4 cells were cultured with the preincubated EVs in RPMI containing no FBS for 2 days. Then, MOLT4 cells were washed with PBS and plated at a density of 500 cells/well in 96 well-plate in phenol-red free RPMI containing no FBS together with 5000 LNCaP cells. After 2 days, cytotoxicity was assayed using CytoTox 96 non-radioactive cytotoxicity assay (Promega, Madison, WI, USA) and absorbance was measured at 490 nm. Experiments were done in quadruplicate. The results are shown as mean ± SD (*n* = 4). Statistical analysis was carried out using two-sided Student’s *t*-test. **P < 0.01.

## Electronic supplementary material

Supplementary figure legends

Supplementary Figure S1

Supplementary Figure S2

Supplementary Figure S3

Supplementary Figure S4
