# Supplementary material for: Extracellular vesicle-mediated EBAG9 transfer from cancer cells to tumor microenvironment promotes immune escape and tumor progression
Source: Oncogenesis. 2018 Jan 24;7(1):7. doi: 10.1038/s41389-017-0022-6 (PMC5833691; doi:10.1038/s41389-017-0022-6)
Supplement: Supplementary file 1 — Supplementary figure legends [file 41389_2017_22_MOESM1_ESM.doc]

**SUPPLEMENYARY FIGURE LEGENDS**

**Supplementary Figure S1. EBAG9 silencing decreases migration of prostate cancer cells and modulates EMT-related gene expression.** (**A**) siEBAG9 #2 inhibited migration of LNCaP cells. Cells transfected with siEBAG9 #2 or siControl were seeded on the upper chamber and migrated cells were stained after 48 h. Representative images are shown. Scale bar, 20 μm. Effect of siEBAG9 #2 on the migration of LNCaP cells, evaluated by counting migrated cells in 5 microscopic fields at least. Data are shown as mean ± SD (*n* = 5). (**B**) Effect of siEBAG9 #2 on EMT-related gene expression in LNCaP cells. qRT-PCR analyses of *VIM*, *SNAI1*, *SNAI2* and *EBAG9* mRNA were performed using RNAs prepared from LNCaP cells treated with siEBAG9 #2 or siControl. The results are shown as mean ± SD (*n* = 3). **P* < 0.05; ***P* < 0.01 (two-sided Student’s *t*-test). (**C**) EBAG9 siRNAs decrease vimentin protein in LNCaP cells. Western blot analysis of VIM was performed using cell lysates prepared from LNCaP cells transfected with siEBAG9 #1 and #2, or siControl.

**Supplementary Figure S2. Migration of DU145 prostate cancer cells is decreased by EBAG9 knockdown and increased by treatment with extracellular vesicles (EVs) derived from LNCaP-EBAG9 cells.** (**A**) siEBAG9 #1 and #2 inhibits migration of DU145 cells. DU145 cells transfected with siEBAG9 #1, #2 or siControl were seeded on the upper chamber. After 24 h, migrated cells were stained with a Giemsa’s stain solution and counted microscopically. Data are shown as mean ± SD (*n* = 5). Statistical analysis was performed using two-sided Student’s *t*-test. **P* < 0.05; ***P* < 0.01. (Scale bar, 20 μm.) (**B**) siEBAG9 #1 and #2 decreased EBAG9 mRNA expression in DU145 cells. (**C**) EVs from LNCaP-EBAG9 cells stimulate migration of DU145 cells. DU145 cells were incubated with the EVs prepared from LNCaP-EBAG9 and LNCaP-Vector cells. Migrated cells on the lower surface of filters were counted in 5 or more microscopic fields. Data are shown as mean ± SD (*n* = 5). Statistical analysis was performed using two-sided Mann–Whitney U test. **P* < 0.05.

**Supplementary Figure S3.** **EBAG9 overexpression increases vimentin protein in LNCaP cells.** Western blot analysis of VIM was performed using cell lysates prepared from LNCaP-EBAG9 #4 and #6 and LNCaP-Vector #3 and #5 cells.

**Supplementary Figure S4. TM9SF1 silencing decreases cancer cell migration and modulates EMT-related gene expression.** (**A**) siTM9SF1 #2 inhibited LNCaP cell migration. Cells transfected with siTM9SF1 #2 or siControl were seeded on the upper chamber. After 48 h, migrated cells were stained. Representative images are shown. Scale bar, 20 μm. Data are shown as mean ± SD (*n* = 5). (**B**) Modulation of EMT-related gene expression in LNCaP cells treated with siTM9SF1 #2. qRT-PCR analyses for *VIM*, *SNAI1*, *SNAI2* and *TM9SF1* mRNA were performed using RNAs prepared from LNCaP cells treated with siTM9SF1 #2 or siControl. Data are shown as mean ± SD (*n* = 3). **P* < 0.05; ***P* < 0.01 (two-sided Student’s *t*-test).
