# Supplementary figures and images for: Extracellular vesicle-mediated EBAG9 transfer from cancer cells to tumor microenvironment promotes immune escape and tumor progression
Source: Oncogenesis. 2018 Jan 24;7(1):7. doi: 10.1038/s41389-017-0022-6 (PMC5833691; doi:10.1038/s41389-017-0022-6)

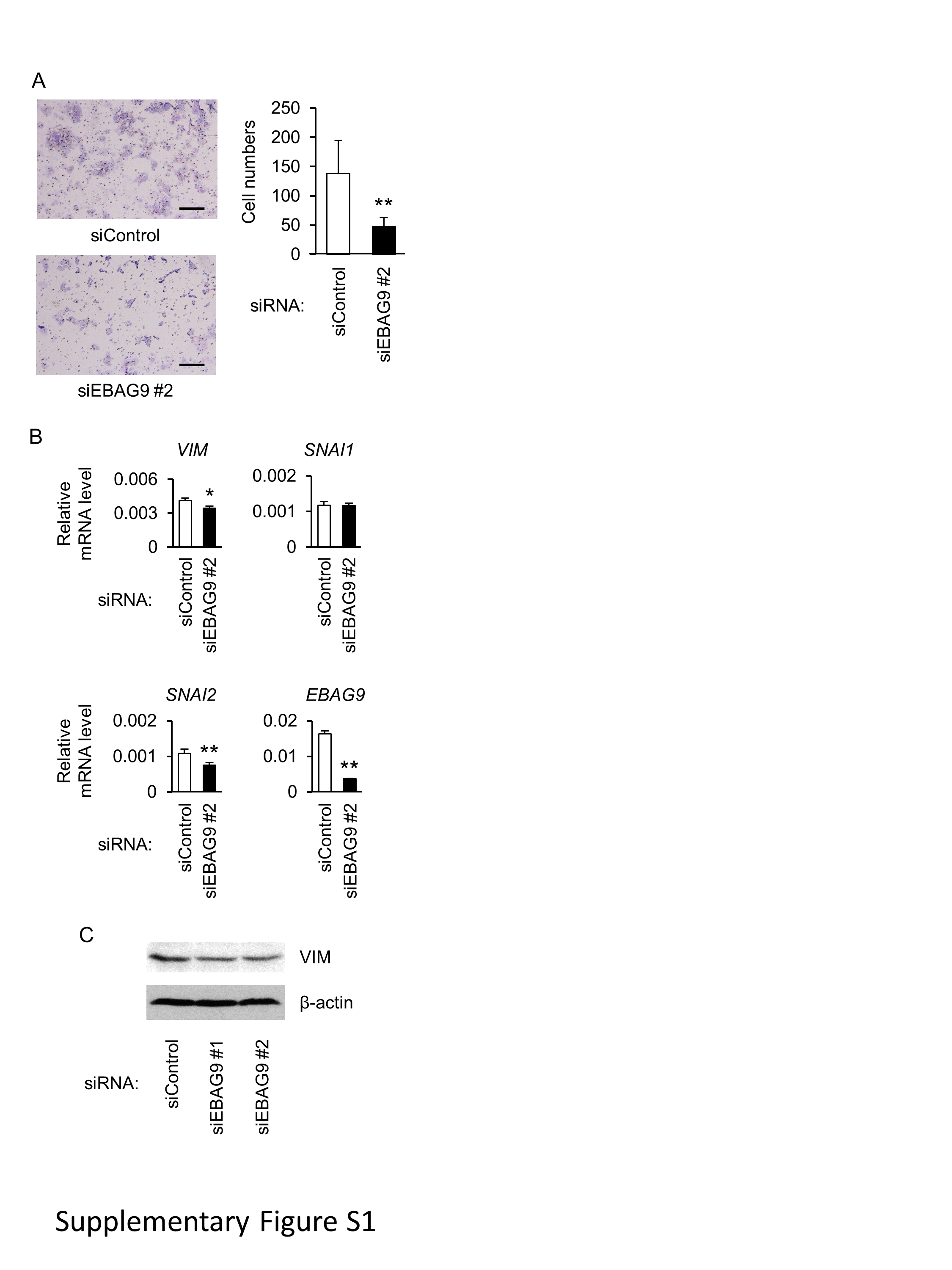

Supplement: Supplementary file 2 — Supplementary Figure S1 [file 41389_2017_22_MOESM2_ESM.tif]

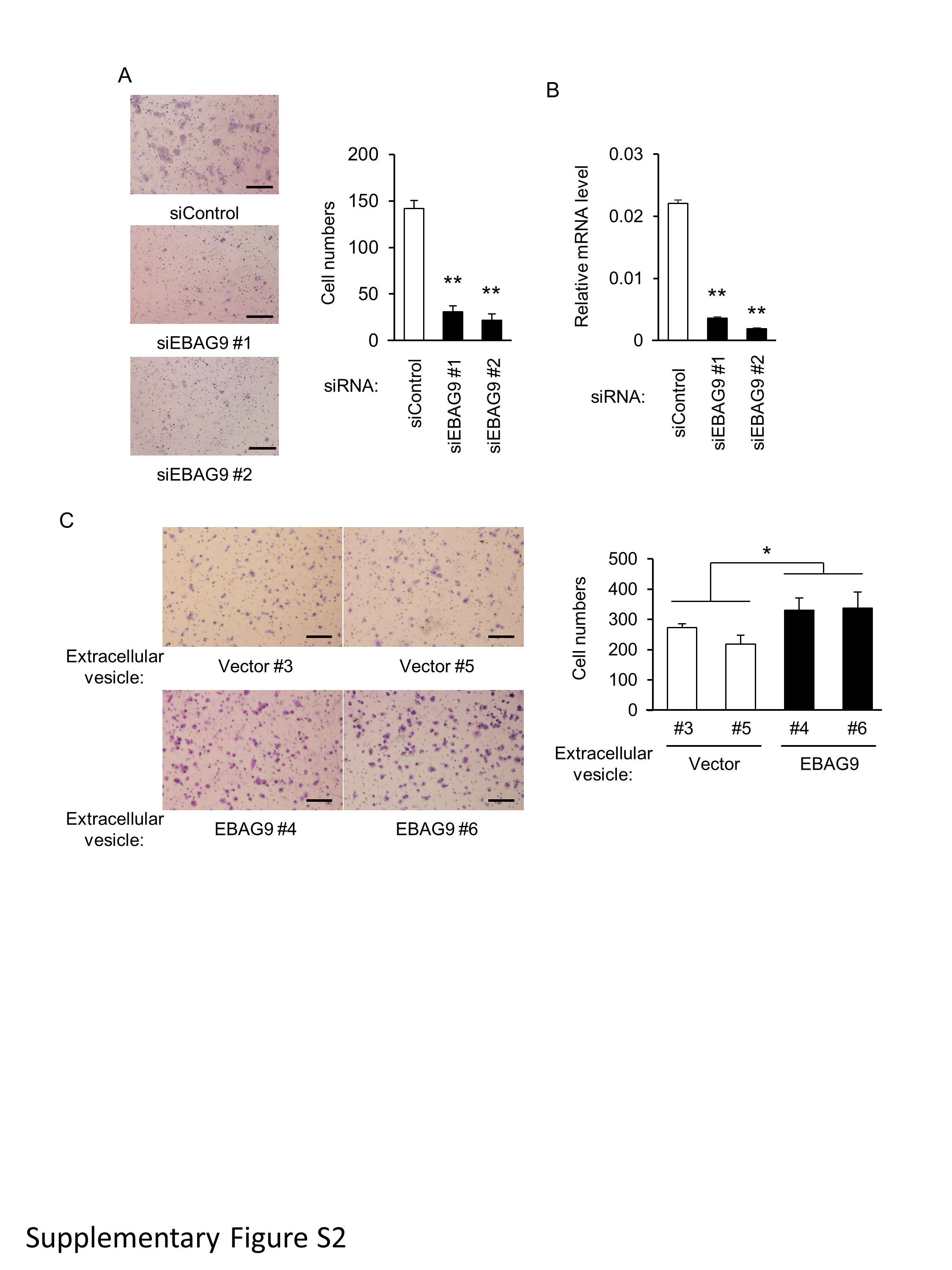

Supplement: Supplementary file 3 — Supplementary Figure S2 [file 41389_2017_22_MOESM3_ESM.tif]

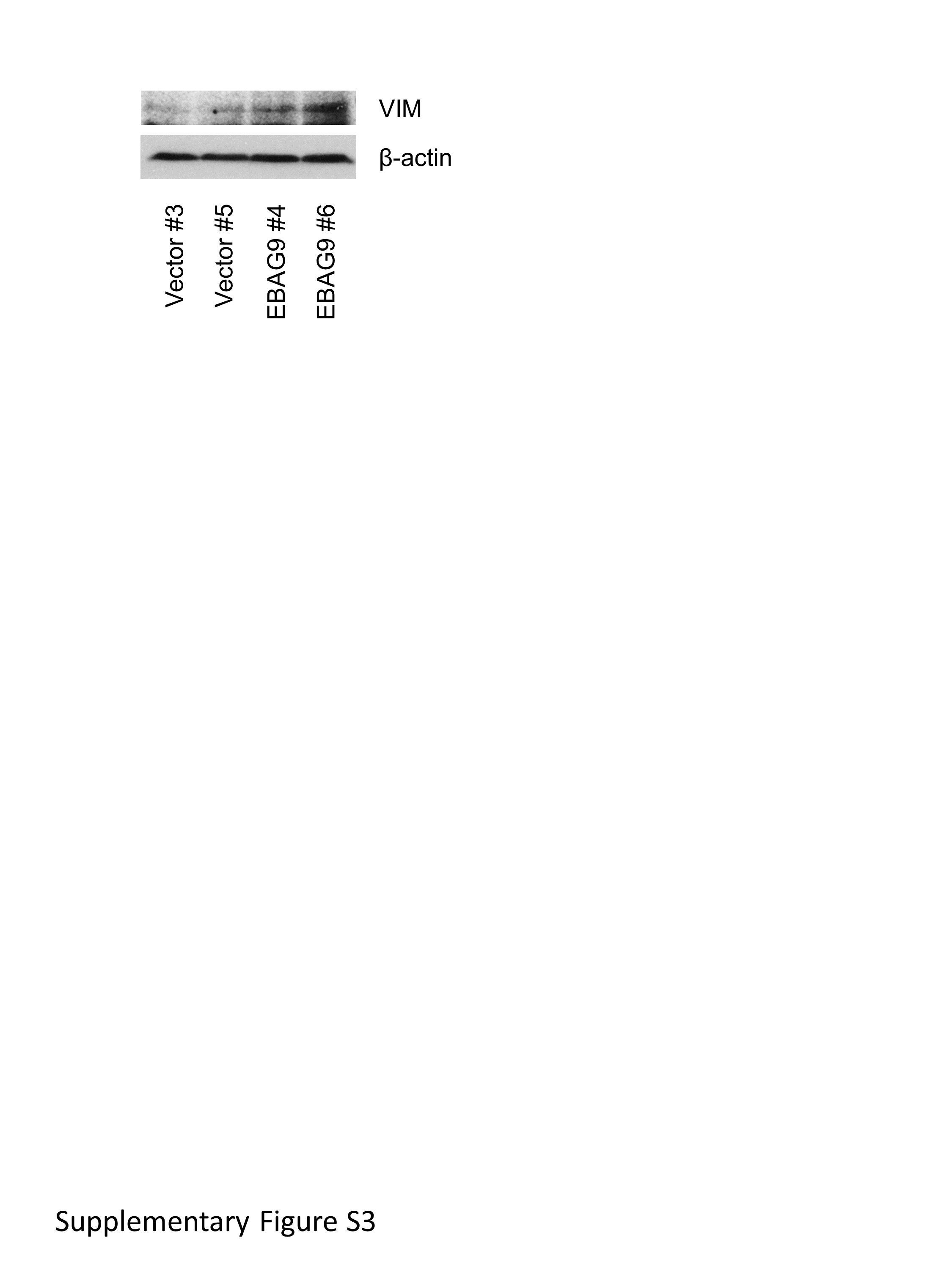

Supplement: Supplementary file 4 — Supplementary Figure S3 [file 41389_2017_22_MOESM4_ESM.tif]

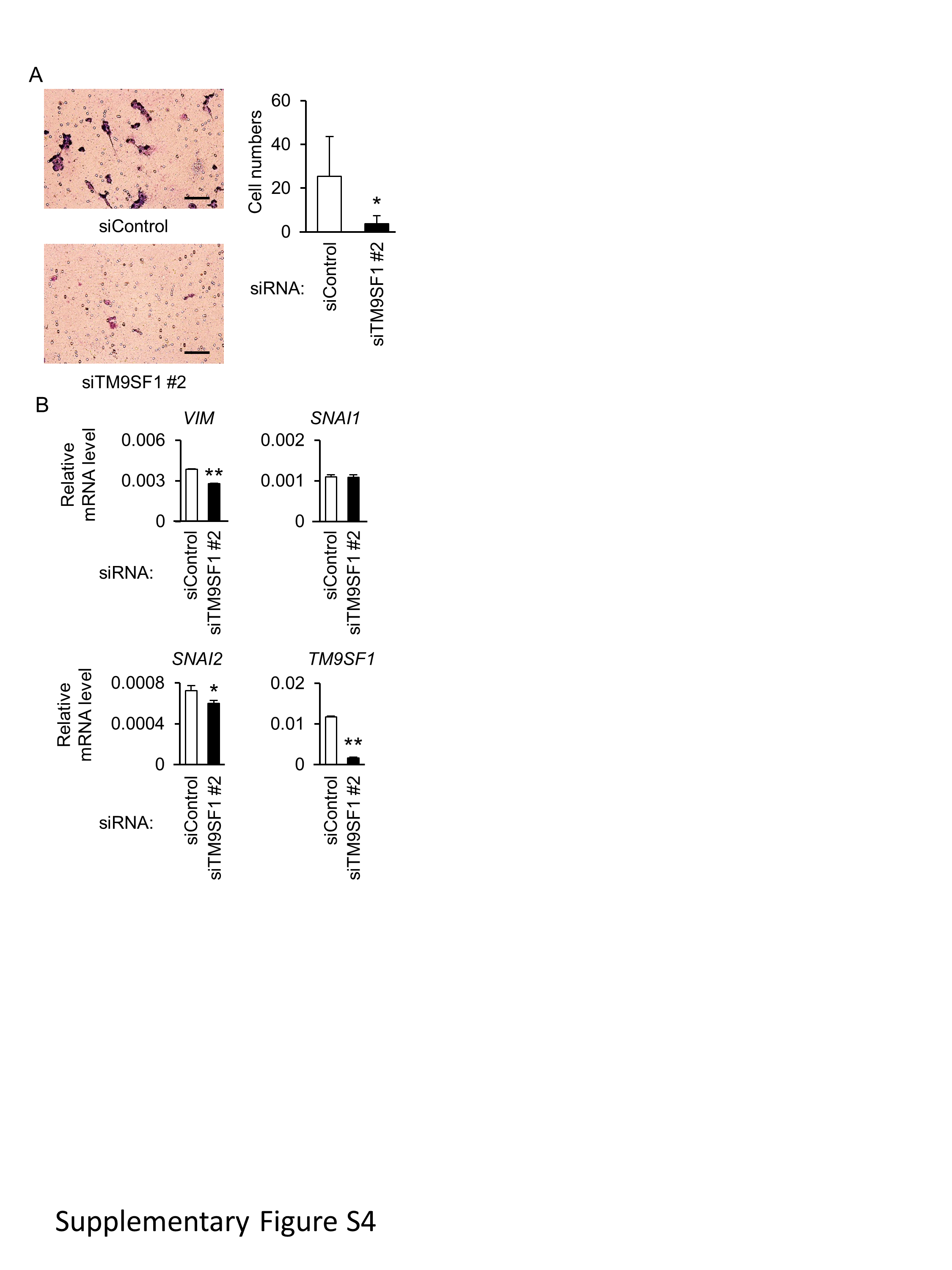

Supplement: Supplementary file 5 — Supplementary Figure S4 [file 41389_2017_22_MOESM5_ESM.tif]
